# Feasibility of implementing public-private mix approach for tuberculosis case management in Pokhara Metropolitan City of western Nepal: a qualitative study

**DOI:** 10.3389/fpubh.2023.1132090

**Published:** 2023-05-24

**Authors:** Sushila Baral, Rajesh Kumar Yadav, Dipendra Kumar Yadav, Sujan Babu Marahatta, Yadunath Baral, Khim Bahadur Khadka, Sanjay Kumar Thakur, Srijana Paudel, Prabin Sharma, Sony Pandey, Kusum Shrestha, Naveen Prakash Shah, Laxman Basaula, Amar Nagila, Roshan Kumar Mahato, Chhabi Lal Ranabhat

**Affiliations:** ^1^Center for Mental Health and Counselling Nepal (CMC Nepal), Kathmandu, Nepal; ^2^Department of Public Health, Manmohan Memorial Institute of Health Sciences, Kathmandu, Nepal; ^3^Save the Children International, Kathmandu, Nepal; ^4^Department of Public Health, School of Health and Allied Sciences, Pokhara University, Pokhara, Nepal; ^5^Department of Orthopedics, Pokhara Academy of Health Sciences, Pokhara, Nepal; ^6^Health Directorate, Minstry of Health and Population (MoHP), Gandaki Province, Pokhara, Nepal; ^7^National Tuberculosis Control Center, Thimi, Bhaktapur, Nepal; ^8^Department of Medicine, Pokhara Academy of Health Sciences, Pokhara, Nepal; ^9^Provincial Health Training Center, Gandaki Province, Pokhara, Nepal; ^10^Provincial Government, Health Office, Damauli, Tanahun, Nepal; ^11^Department of Medical Microbiology, School of Health and Allied Sciences, Pokhara University, Pokhara, Nepal; ^12^School of Medical Sciences, Kathmandu university, Dhulikhel, Nepal; ^13^Global Center for Research and Development, Kathmandu, Nepal

**Keywords:** tuberculosis, private public mix approach, Nepal, case finding, qualitative

## Abstract

**Background:**

The Public-Private Mix (PPM) approach is a strategic initiative that involves engaging all private and public health care providers in the fight against tuberculosis using international health care standards. For tuberculosis control in Nepal, the PPM approach could be a milestone. This study aimed to explore the barriers to a public-private mix approach in the management of tuberculosis cases in Nepal.

**Methods:**

We conducted key informant interviews with 20 participants, 14 of whom were from private clinics, polyclinics, and hospitals where the PPM approach was used, two from government hospitals, and four from policymakers. All data were audio-recorded, transcribed, and translated into English. The transcripts of the interviews were manually organized, and themes were generated and categorized into 1. TB case detection, 2. patient-related barriers, and 3. health-system-related barriers.

**Results:**

A total of 20 respondents participated in the study. Barriers to PPM were identified into following three themes: (1) Obstacles related to TB case detection, (2) Obstacles related to patients, and (3) Obstacles related to health-care system. PPM implementation was challenged by following sub-themes that included staff turnover, low private sector participation in workshops, a lack of trainings, poor recording and reporting, insufficient joint monitoring and supervision, poor financial benefit, lack of coordination and collaboration, and non-supportive TB-related policies and strategies.

**Conclusion:**

Government stakeholders can significantly benefit by applying a proactive role working with the private in monitoring and supervision. The joint efforts with private sector can then enable all stakeholders to follow the government policy, practice and protocols in case finding, holding and other preventive approaches. Future research are essential in exploring how PPM could be optimized.

## Introduction

Tuberculosis is a highly contagious infectious disease that is one of the world’s deadliest. Globally, about 10 million people developed the disease, and 98% of the world’s registered TB cases were in low-and middle-income countries (LMICs) (1). In Nepal, around 40% of Nepalese participants in the End TB program did not seek TB care and treatment, according to Nepal’s 2018 national TB prevalence survey (2). Approximately 15 people die by tuberculosis, and over 180 people become ill with this preventable and curable disease in Nepal (2).

Tuberculosis control program was systematically started about 6 decades ago and there was a triple agreement between Government of Nepal, World Health Organization and UNICEF (3). Since that time, there has been historic progress toward the tuberculosis control program in Nepal. Primary health care to UN sustainable development goal, tuberculosis control is one of the important target and quality service is still challenge in Nepal (4, 5). Only state approach is not sufficient and utilization of private sector and community participation is essential to control TB control program (6). The Public-Private Mix (PPM) approach is a strategic initiative with the goal of enlisting all private and public health care providers in the fight against tuberculosis while adhering to international health care standards. It is a globally effective intervention strategy. Public-Private Mix has played a significant role in tuberculosis control, significantly contributing to case detection, diagnosis, and treatment (7). A public-private mix (PPM) model for directly observed therapy short course (DOTS), involving a partnership between the government and the private health sector, plays a critical role in accelerating progress in TB control. Private sector contributed 19, 18 and 23 percent for the diagnosis and treatment of TB cases in 2015, 2016 and 2017, respectively, in Nepal (6). An ongoing challenge is identifying hidden cases and ensuring that identified tuberculosis patients complete treatment according to protocol. When the PPM approach is not implemented, a presumptive TB patient visits a private service provider that is not affiliated with the National Tuberculosis Programme (NTP) and undergoes a slew of unnecessary diagnostic tests before receiving the correct diagnosis and anti-TB treatment. When the PPM approach is implemented, the patient receives a free sputum microscopy test and, if diagnosed with TB, is given free anti-TB drugs.

Previous studies mostly focus on prevalence (8), multiple factors associated with diagnosis, treatment status (9, 10), health seeking behaviors (11), knowledge and awareness level regarding tuberculosis in Nepal. Studies identified various barriers such as public sector factors, private sector system factors, program implementation factors, and policy for private public mixed method (12, 13). TB burden and case notification rates in Nepal vary across provinces and nations over time. It is critical to identify actual barriers in the context of Nepal. Approximately 69,000 people diagnosed with tuberculosis (TB) in 2018, but the incidence was 1.6 times higher; approximately 117,000 people are living with TB, and 30,000 cases were missed to detect (2).

Presumptive TB patients are more likely to visit private hospitals for identification and diagnosis of TB, but the majority of private hospitals do not report cases to government health systems. If cases diagnosed in private hospitals were notified and enrolled in the government health system, the target cases for the national tuberculosis control program could be met to some extent (14). There is still challenge to find out the new case in rural, urban slums area and HIV co infection in tuberculosis control program and appropriate utilization of private sector with community participation would be the opportunity (6). The study’s goal was to identify the barriers to implementing the private-public mix approach to finding tuberculosis cases, which would be useful for policymakers and program managers planning to implement the PPM approach. Based upon these insights, collaboration should be done with the private sector, and jointly supportive monitoring and supervision to identify gaps in the private sector would help in case finding. Effective training and orientation programs for all practitioners, including those in the private sector, will help the tuberculosis program, the recording and reporting system, and treatment outcomes.

## Materials and methods

### Study design and participants

This was a qualitative study conducted in Pokhara Metropolitan City with various levels of health personnel (pharmacists, medical recorders, medical officers, and policymakers) that adhered to a standard consolidated criteria for reporting qualitative studies (the COREQ guideline) (15). The study investigated data collection techniques based on the phenomenological approach in qualitative research to investigate the barriers to implementing the PPM approach for TB case findings, which allows people who are a part of it or have first-hand experience to describe the phenomenon of interest while avoiding any predesigned framework (16). All members of the research team have extensive experience in quantitative and qualitative studies, as well as working in the tuberculosis sector and providing TB-related health services.

A variety of participants were interviewed using key informant interviews (KIIs). The study’s purpose, procedure, and rationale were explained to all enrolled participants. Participants had worked in a tuberculosis program for more than 2 years. Participants who agreed to participate were informed about the procedure and asked to sign a written informed consent. Participants were informed that they could withdraw from the study at any time during the interviews for any reason.

### Study site

**Figure 1:**
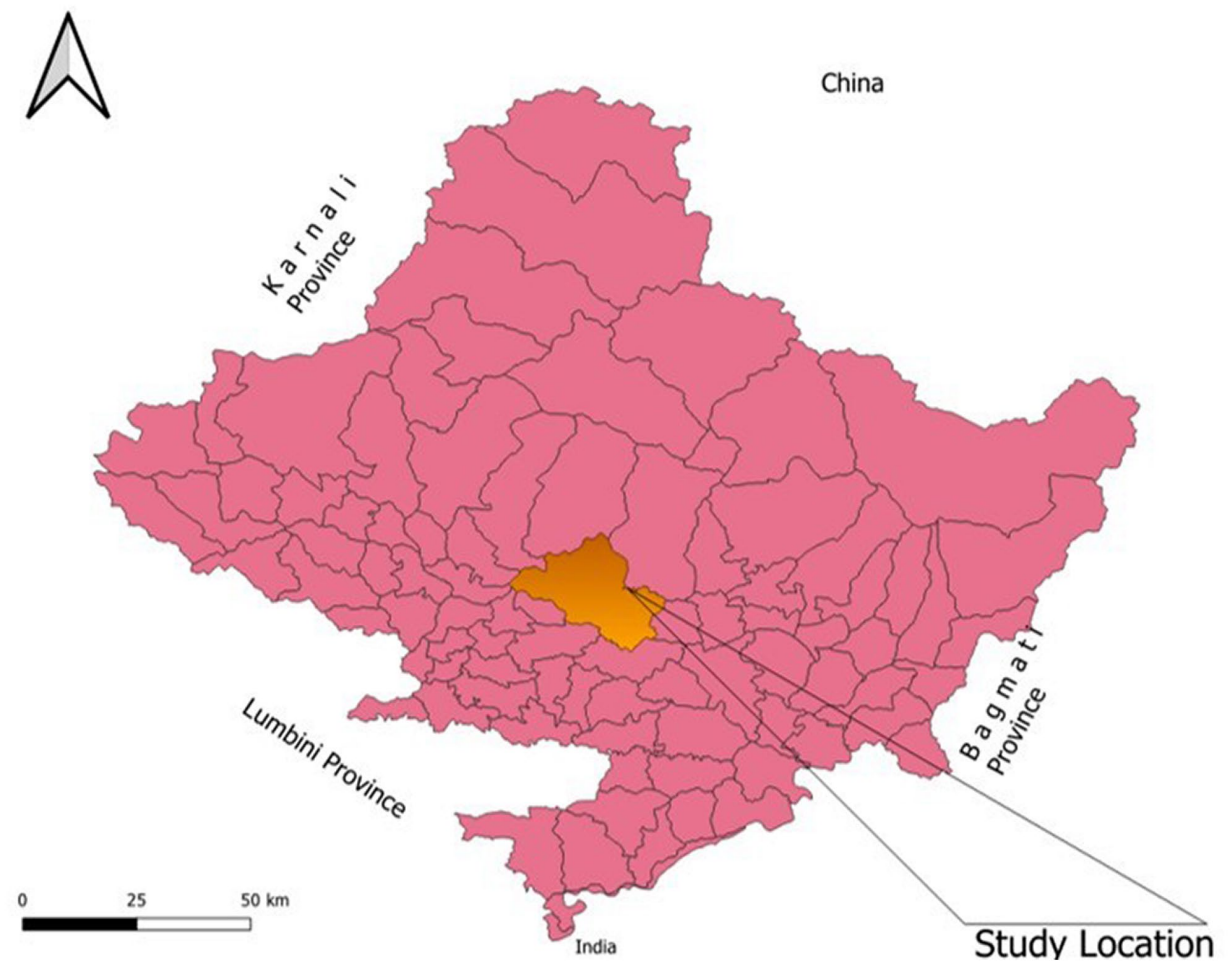
Study site.

As the country moves toward a federal system, Nepal is divided into seven provinces. Nepal’s municipalities are divided into metropolitan, sub-metropolitan, and rural areas. Nepal has altogether six metropolitan cities (Kathmandu, Pokhara, Bharatpur, Biratnagar, Lalitpur, and Birgunj), and the PPM approach is implemented in all of them among which Pokhara Metropolitan City was randomly selected. The study was carried out in the Pokhara Metropolitan City (Figure 1), which lies in Gandaki Province. About 20 health facilities were enrolled in the study. Four PPM implemented pharmacies, two PPM implemented polyclinics, two PPM implemented hospitals, two PPM intervention linkage but not working pharmacies, two government hospitals, and two PPM not implemented hospitals were included. Two representatives from the TB Program Implementation Government Body and four tuberculosis program supervisors were also enrolled in the study.

We simply selected the participants who have been involved in TB diagnosis, treatment follow up or possibly involved in future based on case TB caseload. The participants were the representatives from directly involved in TB control program so that they could explore the barriers and hindering factors in PPM approach and ultimately recommend for policy departure to government agencies.

### Data collection and interview guide

Face-to-face interviews were conducted between June and August 2022. All interviews were audio-recorded. Participants participated alone in the interviews where there is a peaceful environment to avoid noise. Four researchers (SP, KS, SrP, and YNB) conducted the interviews. Before conducting interviews, they were trained in qualitative research by research experts.

Respondents were approached for interviews aligning with the objective of the study. Individual respondents were interviewed by interviewers who introduced themselves and briefed them on the study (its objectives and procedures, as well as the potential benefits and harms of participation). The interviews were conducted in the local (Nepali) language and lasted for around 20 to 40 min. The interviewer also noted the emphasis placed by the interviewees during the interview, which aided the data analysis.

None of the candidates refused to participate, and no repeat interviews were carried out. Based on the principles of “data saturation,” the sample size for this study was deemed sufficient when no new data or themes emerged from further interviews.

### Data analysis

**Figure 2:**
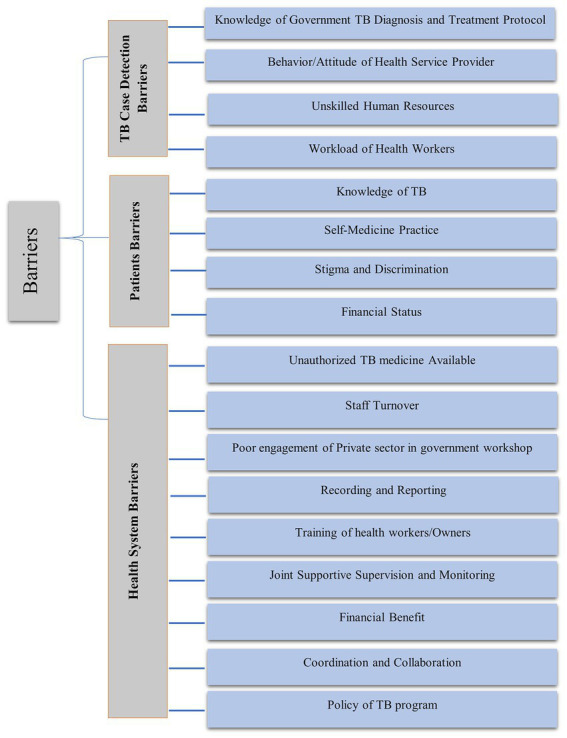
Barriers to implement PPM.

All audio-recorded interviews and discussions, together with the field notes taken during the interviews, were transcribed and translated into the English language. A pre-designed codebook guided the initial data analysis. Three researchers (SB, RKY, and PS) coded the data independently. A thematic analysis of the transcripts was conducted by the first author, and samples from the transcripts were cross-checked by DKY, SKT, NPS, KBK, and LB. An inductive approach to thematic analysis was undertaken to ensure all themes were identified. Final themes were discussed among the authors and categorized into main themes and sub-themes based on their relevance to the research question of this study. The final themes relevant to the research question are broadly categorized into A: TB Case Detection Barriers; B: Patient-Related Barriers; and C: Health System-Level Barriers (Figure 2).

### Ethical statement

Ethical approval was taken from the Institutional Review Committee of Pokhara University Research Centre (PU-IRC 43/077/078). Written informed consent was obtained from the participants for the publication of any potentially identifiable images or data included in this article. The research was explained to respondents and verbal consent was taken from all respondents prior to conducting the interview. Participants were informed that confidentiality would be maintained during the research. They were also made aware that their participation was voluntary and could withdraw their participation at any point without any consequences.

## Results

There are 33 HFs in Kaski District that are linked in a private-public mixed approach to increase TB case notification and reduce missing TB cases in the Gandaki Province.

### Characteristics of participants

The 20 participants included 14 from private clinics, polyclinics, or hospitals where the PPM approach was used, 2 from government hospitals, and 4 policymakers (Table 1). Participants who had more than 2 years’ experience in TB programs were interviewed. The ages of the participants ranged from 23 to 57 years; the mean age was 32.85 years, and two-thirds of them were male.

**Table 1:** Socio-demographics of participants in the study.

Participant code	Institutions	Working experience in TB	Education level	Working organization
P1	PPM Implemented pharmacy	3 Years	Diploma in pharmacy	Private clinic
P2	PPM Implemented pharmacy	5 years	Health assistant	Private clinic
P3	PPM Implemented pharmacy	3 years	Health Assistant/Bachelor of public health	Private clinic
P4	PPM Implemented pharmacy	4 Years	Diploma in Pharmacy	Private clinic
P5	PPM Implemented polyclinic	2 years	MSc. Nursing	Private poly clinic
P6	PPM Implemented polyclinic	2 Years	Diploma in pharmacy	Private poly clinic
P7	PPM Implemented hospital	5 Years	Bachelors in education	Private hospital
P8	PPM Implemented hospital	2 Years	Master in science in microbiology	Private hospital
P9	PPM Intervention linkage but not working	2 Years	Diploma in dental	Private hospital
P10	PPM Intervention linkage but not working	6 years	Community medicine assistant	Private hospital
P11	PPM not Implemented hospital	4 Years	MD-internal medicine	Private hospital
P12	PPM not Implemented hospital	6 Years	MD-internal medicine	Private hospital
P13	Government hospital	3 Years	MD-internal medicine	Government hospital
P14	Government hospital	5 Years	MD-internal medicine	Government hospital
P15	TB Program implementation government Body	10 Years	MD in Pulmonology, critical care and sleep medicine	Government hospital
P16	TB Program implementation government Body	25 Years	Lab Technician	Government hospital
P17	PPM Program supervisor	5 Years	Health assistant	Private organization
P18	PPM Program supervisor	2 Years	Health assistant	Private organization
P19	PPM Program supervisor	2 Years	Health assistant	Private organization
P20	PPM Program supervisor	5 Years	Health service management	Private organization

### Major themes

#### Case detection barriers

##### Knowledge of TB diagnosis system and treatment protocol

Private service providers reported that they do not have updated knowledge of the government treatment protocol as private practitioners are not involved in training and orientation programs; they do not know about the PPM approach.

*“Government service providers are provided with training and orientation annually to be updated on the strategy and guidelines, but we private practitioners were not involved in any such training; whatever updated knowledge we had was through surfing the internet; we get only some updates.” (PPM-Implemented Pharmacy)*

*“The presence of doctors for 24 hours isn’t possible in any pharmacies or polyclinics; we have some physicians “on call.” "Many patients were missed for proper diagnosis due to the lack of regular presence of skilled physicians.” (PPM Supervisor)*

Most of the private service providers were found to be unaware of the implementation of the PPM approach, treatment regimen, protocol, and referral system in Nepal.

*“I am new on this pharmacy and had not got any orientation and training regarding PPM approach. Sorry, I am unknown in this matter” (PPM implemented pharmacy)*

##### Behavior/attitude of the private health service provider

Most of the service providers conveyed that they had a busy schedule and needed to work in many pharmacies and hospitals, so they only visited when there was a call. They give medicines based on the signs and symptoms without using any diagnostic tools.

*“Interaction with the same patient is rare; we do prescribe medicine according to their signs and symptoms, and if it is presumptive TB patients, we refer them to a tuberculosis treatment center and don’t do follow-up as we are not available for 24 hours, so whether they visited or not, we don’t have time to care.” (PPM-Implemented Pharmacy)*

Another perspective from government stakeholders reflected that the PPM model has been practiced in many areas but that there is an implementation problem because private practitioners are not interested in this model in reality. Private service providers prioritized profit over service. They had an attitude as follows:

*“If they refer patients to the government DOTS center, they will look after the patient at no cost for the whole six months and they will not come back to them; another reason for not having the interest of doctors is that during the one-time follow-up, private service providers prescribe medicine to the patients." The patient should pay the ticket fee the next time he or she comes in for a follow-up appointment. In this way, private service providers earn money. This is why patients do not come to government service centers. This is also one of the reasons for patient dropout and an increase in MDR-TB cases.” (TB Program Implementation Government Body)*

##### Workload of health workers

Multitasking by the same person and excessive workload at the workplace make the program profligate. Orientation regarding the program is a must for the staff, but due to their workload, they are unable to participate in orientation and training sessions. Physicians have busy schedules as they are working on call and have no time to detect presumptive TB cases. When they are diagnosed, insufficient time for counseling is directed toward follow-up.

*“I've been working in this polyclinic for 6 years, and we two are here; some medical officers are kept on call; we have to look after all the clients, and if any TB suspected patient visits our health institution, we ask them to visit the tuberculosis center, and afterwards we don't do follow-up because we don't have enough time; some of them refuse to go, so we give them medication.” (PPM hospital implementation)*

#### Patients barriers

##### Knowledge of TB and self-medicating practice

Service providers at DOTS centers reported that the majority of patients who visit with signs and symptoms of TB have a poor level of knowledge. People were still not responsive; they do not feel comfortable sharing about the disease and do not want to be exposed as TB patients in society, thinking their social prestige will suffer. There is also a need for proper counseling in terms of social mobility.

*“It is very difficult to counsel them about the medication and follow up.” Contracting them repeatedly for follow-up by health workers is viewed as a benefit of health care providers. “They think we have a financial benefit, so we are contacting them, but in reality, we are counseling them for their own benefit and controlling MDR-TB.” (Government Hospital)*

Service providers reported that the majority of the presumptive TB cases were referred to tuberculosis centers for treatment, but they are tenacious in their words that they cannot have TB. They only saw the poor’s disease and refused to accept reality. Patient insisted on cough syrup and anti-cold medicines. Due to a lack of proper counseling and the patient’s misconception that they cannot have TB, they do not want to have a sputum test, which also leads to missing cases.

*“Whenever he found patients with similar signs and symptoms of TB, he counseled them and referred them to the nearest DOTS center, but they didn't visit the center instead going to a side-by-side clinic and taking the medicine,” one of the private service providers said.*

##### Stigma and discrimination

Participants shared their perspectives on the prevailing stigma and discrimination still present in the community. Patients were afraid that their disease condition would be reflected in society, so they went to the clinic secretly and demanded the medicine. They perceived that if it is revealed, their prestige and respect will be diminished.

*“Patients don’t like to travel to a tuberculosis center for medication as they fear that others will know and think negatively about their TB status while visiting the hospital.” “During the time that patients are visiting a TB center regularly to take medicine, other people who have misconceptions about TB may behave disrespectfully and hate the patient; that’s why patients take medicine from a private pharmacy.” (PPM is not used in the hospital.)*

*“Few percent of patients belong to those groups who do not want other people to know that they are TB patients or their TB status, because of which they decide to visit a private clinic or pharmacy for the medicine and take it for 2-3 months before returning home. Those patients are unaware that they are not being treated in accordance with government protocol, and they experience relief from high doses of medicine after a few months but do not receive full course treatment. These are the same people who will develop MDR.” (TB Program Implementation Government Body)*

##### Financial status

Presumptive TB cases are referred to the polyclinic (defined as hub centers) by the pharmacy, and patients had to pay the OPD charge of the clinic, which added financial burden to the family.

*"‥mostly those people who can't afford the OPD charge of hospitals visit here for check-up; we check for their fever and cough; thinking slogan of "more than 7 days of continuous cough might be TB," we refer them to a tuberculosis center, but they denied to visit; we ask them to come again for follow-up and diagnosis, but they refuse and just want to take medicine for their fever and cough." (PPM-Implemented Pharmacy)*

The transportation cost to visit government health facilities has also created a barrier to the implementation of the PPM approach and patients’ access to health services for diagnosis.

### Health system barriers

#### Staff turnover

Staff turnover in pharmacies is also a source of concern. The PPM model program orients one staff of PPM-implemented clinics, but after some time, they leave the job, leaving only coordination and counseling to the pharmacy staffs.

*"As the pharmacy is linked to the PPM approach, we counsel and train the staff of that clinic; define their roles and working modality of PPM through one-on-one counseling and engaging them in the program; they start their roles but after some time they leave the job and training another person to work smoothly is hampering the program. If we engage the main pharmacy owner in training and orientation, it might be beneficial."*

### Poor engagement of private sector in government workshop

Majority of the private practitioners grumbled about the involvement of the private sector in government programs. The government conducts different programs, training, and orientations just focusing on government health facilities.

*"We are always deprived of getting information." We don’t know about the PPM approach, though I worked in different polyclinics and private hospitals. "Being a physician, if I had been oriented, then I would circulate the program modality where I do work also; it is necessary to involve all private practitioners, at least all physicians, in this type of program." (PPM is not used in the hospital.)*

### Trainings of health workers/orientation of TB programs to owners

Most of the practitioners of polyclinics, pharmacies, and private hospitals where PPM is linked and implemented are concerned with training and orientation regarding TB. Involving private practitioners to update the information is crucial to implementing the approach in an effective way. Involving owners along with the two staff members is needed as there is a high rate of staff turnover. Staff who will retain more in the workplace should be involved in the orientation process.

*"We do know the protocol of Nepal through the curriculum (although there is not much more in the curriculum about all the implemented programs) and induction training, but those medical officers who had completed their study in foreign countries and are now practicing in the private sector (were not provided with induction trainings either) are not well known about all the programs and protocol; how can they know about PPM and refer to it?" one of the government doctors revealed.*

### Recording and reporting system

Recording and reporting are seen as tedious jobs, but they are mandatory for the public-private mix approach to increased case notification. Due to a lack of staff and the lack of forms or formats, there was no reporting in the pharmacy, whereas in private hospitals, due to a busy schedule, staff did not provide time for recording. Mandatory recording and reporting systems at government facilities, on-site coaching, involving the private sector in coordination meetings, and timely monitoring and supervision by government stakeholders would lead to proper recording and reporting. It is extremely difficult to record and report on mobile applications.

*"In the government sector, there is a separate focal person for recording and reporting, but in our workplace, a single person has to do multiple tasks, so implementing another program (the public-private mix approach) will add burden in our workplace." (PPM is not used in pharmacies.)*

*"There is no medical recorder in hospitals, and doctors have no time to register all the records." "The problem is the same in the pharmacy too."*

### Joint supervision and monitoring

Joint monitoring and supervision are crucial to implementing and sustaining the program. Supervision and monitoring from government stakeholders will aid the private sector in working in an effective and efficient way. Viewing records and reports and doing onsite coaching on revised tools, the treatment regimen is lacking. There is a lack of sensitization and awareness about the program’s ability to run effectively.

*"Staff from DDA's health directorate should have a monitoring visit to private clinics and hospitals and should be brief in recording and reporting, provide forms and formats, and all health service providers should be kept under one umbrella; then only the concept of a public-private mix will be effectively implemented." (PPM hospital implementation)*

### Financial benefit

Tuberculosis medicine and laboratory services are provided free by the government, whereas patients need to purchase them from pharmacies. Referring clients from the private sector or pharmacies to the public sector and referring presumptive TB cases from clinics to hub centers ultimately affect their financial productivity. Furthermore, some clinics treat patients for free before referring them to a polyclinic where patients must pay a high OPD charge, lowering their prestige.

*"We are here for easy access to health services for all people as well as for profit, so referring the suspected TB patients to other polyclinics (hub centers) will diminish our business; we can’t lose our patients and refer them to another clinic." (PPM Intervention Linkage Is Ineffective)*

### Coordination and collaboration

Service providers expressed grave concerns about the coordination and collaboration of government sectors with private service providers. Not engaging the private sector in training and orientation programs had made the private sector diminutive and backward. The doctors of the polyclinic were not aware of the PPM approach, and the benefit of it to the health of the presumptive TB patients is lacking. Doctors, pharmacists, and lab technicians all should coordinate with each other.

The public should also be informed about the DOTS program and the availability of free medicine.

According to TB policymakers, *“about 30–40% of TB patients are diagnosed in the private sector, but we are unable to bring them into the government TB track system due to weak coordination and a collaborative approach; we must coordinate with the private sector, putting hands on hands, and then only will we be able to achieve the end TB strategy.”*

### TB program policy and strategies

Policies are the framework and guiding documents. Guidelines and strategies exist only on paper; they are not widely disseminated in the community. Celebrating 1 day as “TB Day” is not enough to create awareness. Stigma and discrimination are still prevailing. The government should emphasize more on the PPM approach and the FAST strategy.

*"Thousands of pharmacies are there in the province; is it possible to orient or train them on the PPM approach and stop the haphazard use of treatment and the referral system through monitoring? The best way to control the current situation is to ban the TB medicines in the private sector through DDA." Otherwise, incentives should be provided to the clinicians for the whole six months, and programs should be run.*

The TB program implementation body was concerned about monitoring and evaluating the private sector. *“If we are adopting the PPM approach, we must monitor the private sector too through coordination with the drug department and others, monitor the recording and reporting systems, update them on formats, and if they are not following the rules, they must be charged, and those who had good performance must be rewarded.”*

*"However, the GoN initiated and regulated malaria drugs; similarly, TB medicines should be banned in private health facilities, so that people will visit government health facilities and receive free treatment; people do not visit and complete a complete regimen or treatment because they cannot afford the cost of medicine; referring all cases from private to public, they will be tracked and traced in the government TB program, and we will be able to achieve the target."*

## Discussion

This is qualitative study to explore the barriers in PPM in TB control program particularly case findings, case holding and preventive measures so that TB case could complete the treatment according to treatment protocol. On the other hand, PPM would contribute to minimize the complications and transmission to another person. The major theme was categorized under case detection, patients’ behaviors and health system barriers. Private health sectors who involved in TB control program were interested to their profit where government organization/personnels with office time and need to motivate with social responsibility because TB transmission affects every sector of community and state.

Our study discovered that private practitioners spend a small amount of time in polyclinics and private hospitals; they usually visit clinics “on call”; additionally, there are a limited number of staff and the need to multitask, which is similar to Lucie Blok’s study (17) which discovered that health workers and managers complained about critical staff shortages, resulting in multitasking and a perception of workload in the workplace (18). Das and Baidya (19) as well as (20) highlights poor knowledge of TB among the tuberculosis patients. Another study of Samal (21) highlights contrast finding which showed good knowledge of TB patients on TB.

Identifying barriers to TB treatment at community health centers revealed that a lack of healthcare providers also had an impact on TB treatment care (22). Individual training will be more difficult to provide when new staff members lack TB management experience (23). The frequent turnover of paramedics and laboratory technicians has been cited as a major source of concern in using private hospitals (12). Because of differences in diagnosis and treatment criteria, there was ineffective external coordination with private healthcare facilities. Coordination with TB stakeholders was difficult in managing TB cases according to national guidelines (23). Another study identified poor recognition of the private sector, in addition to training and orientation, as a barrier to PPM (12). Not all private health care centers have the necessary equipment to diagnose tuberculosis. This can result in incorrect TB diagnosis and treatment. As a result, failures of private centers to refer or report TB patients to government centers have been identified as case-finding and reporting issues (23). Patient confidentiality was cited as the top reasons for not referring patients to RNTCP (12). A study identified five barriers in the TB treatment and monitoring activities: delayed treatment, different TB regimens, inadequate drug monitoring, non-adherence to medication, and difficulties in managing adverse drug reactions (23, 24). Given that public health facilities have significant responsibilities in healthcare, collaboration with private facilities through the PPM program is strongly encouraged. Nonetheless, a study found that PPM at the government health center faced several challenges, including a lack of private sector interest, a lack of exposure to the DOT strategy in the private sector, and insufficient local regulation to engage private sectors. Human resource shortages and external coordination, as described in organizational capacity, impacted the performance of the PPM program at the community level (23). Study of India and Kerala found no good involvement of private sectors toward NTP. Some of the factors identified for poor private sector engagement included: a lack of initiative on the part of the government, a negative attitude among government employees toward the private sector, and power imbalances between government employees and service providers at private clinics/hospitals (12). Another reason given by private sectors for not referring patients to the government system is fear of losing them (12). As a result, private hospitals regard public health as a low priority issue. The private sector is preoccupied with patients and business (12).

Pradipta et al. discovered stigmatization of TB patients in the community, in which the patients felt the community disapproved of them and feared close contact with them (25). For any public-private mix model to work, the government must fulfill its stewardship role with sincerity and responsibility. This allows the partners to develop trust. According to the findings of the study, any flaws in the partners’ liaison lead to numerous misunderstandings. Finally, it is critical to ensure that the patients, who are the primary beneficiaries of this collaboration, are not marginalized (26).

## Conclusion

The study concludes that numerous barriers exist in the way of effective implementation of the PPM approach, including TB case detection barriers such as knowledge of TB diagnosis and treatment, HW attitude, workload, patient-related barriers such as knowledge of TB, self-medication practice, stigma and discrimination, financial status, and health system-related barriers such as staff turnover and poor engagement of the private sector in workshops, trainings, recording, and re-evaluation. Government stakeholders must work together with private sector stakeholders to perform joint monitoring and supervision. Private practitioners should receive training and orientation, and presumptive TB patients should be given adequate time and counseling as well as motivation to visit a government health facility.

### Limitation

This comprehensive finding cannot be generalized to the entire setting. However, the findings demonstrate the possible processes and explore the barriers to implement PPM approach to improve TB case finding.

## Data availability statement

The raw data supporting the conclusions of this article will be made available by the authors, without undue reservation.

## Ethics statement

Ethical approval was taken from the Institutional Review Committee of Pokhara University Research Centre (PU-IRC 43/077/078). Written informed consent was obtained from the participants for the publication of any potentially identifiable images or data included in this article. The research was explained to respondents and verbal consent was taken from all respondents prior to conducting the interview. Participants were informed that confidentiality would be maintained during the research. They were also made aware that their participation was voluntary and could withdraw their participation at any point without any consequences.

## Author contributions

SB, RY, DY, and NS: conceptualization. SB, RY, RM, AN, PS, and SrP: data curation. SB, KS, SoP, YB, and RY: formal analysis. KK, LB, and ST: investigation. DY, SB, RY, SM, RM, and PS: methodology. SB, SrP, NS, KK, and AN: supervision. ST, SB, SM, and RY: validation. ST, SM, KK, and DY: visualization. SB, RY, SrP, LB, and AN: writing – original draft. SB, CR, RY, DY, KK, ST, NS, and RM: writing – review and editing. All authors contributed to the article and approved the submitted version.

## Conflict of interest

The authors declare that the research was conducted in the absence of any commercial or financial relationships that could be construed as a potential conflict of interest.

## Publisher’s note

All claims expressed in this article are solely those of the authors and do not necessarily represent those of their affiliated organizations, or those of the publisher, the editors and the reviewers. Any product that may be evaluated in this article, or claim that may be made by its manufacturer, is not guaranteed or endorsed by the publisher.
