# Supplementary figures and images for: Feasibility of implementing public-private mix approach for tuberculosis case management in Pokhara Metropolitan City of western Nepal: a qualitative study
Source: Front Public Health. 2023 May 24;11:1132090. doi: 10.3389/fpubh.2023.1132090 (PMC10244665; doi:10.3389/fpubh.2023.1132090)

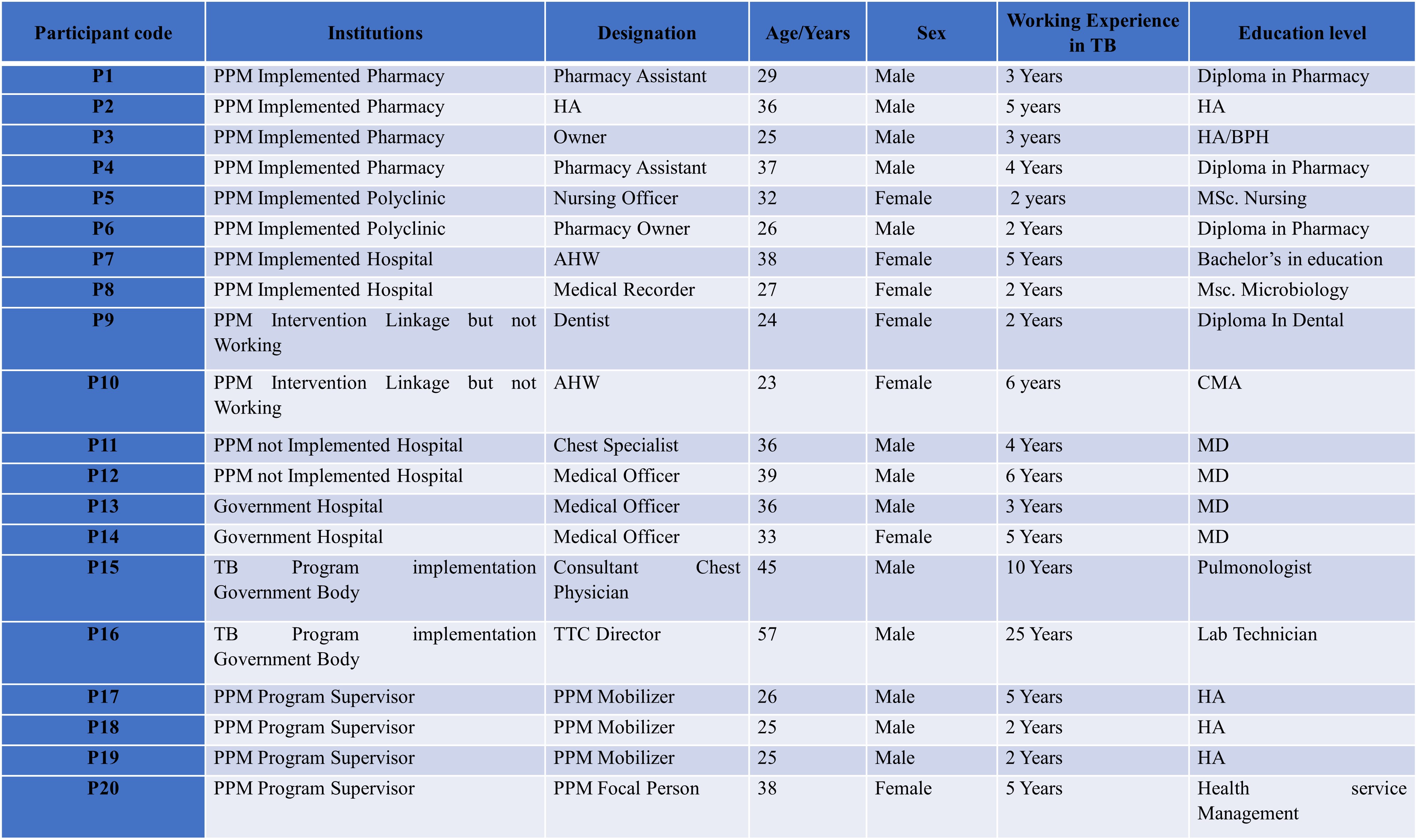

Supplement: Supplementary file 1 [file Image_1.JPEG]
